# Mineralocorticoid Receptors Guide Spatial and Stimulus-Response Learning in Mice

**DOI:** 10.1371/journal.pone.0086236

**Published:** 2014-01-21

**Authors:** J. Marit Arp, Judith P. ter Horst, Sofia Kanatsou, Guillén Fernández, Marian Joëls, Harm J. Krugers, Melly S. Oitzl

**Affiliations:** 1 Center for Neuroscience, Swammerdam Institute for Life Sciences, University of Amsterdam, Amsterdam, The Netherlands; 2 Department of Neuroscience and Pharmacology, Brain Center Rudolf Magnus, University Medical Center Utrecht, Utrecht, The Netherlands; 3 Department of Cognitive Neuroscience, Donders Institute for Brain, Cognition and Behaviour, Radboud University Nijmegen Medical Center, Nijmegen, The Netherlands; Max Planck Institute of Psychiatry, Germany

## Abstract

Adrenal corticosteroid hormones act via mineralocorticoid (MR) and glucocorticoid receptors (GR) in the brain, influencing learning and memory. MRs have been implicated in the initial behavioral response in novel situations, which includes behavioral strategies in learning tasks. Different strategies can be used to solve navigational tasks, for example hippocampus-dependent spatial or striatum-dependent stimulus-response strategies. Previous studies suggested that MRs are involved in spatial learning and induce a shift between learning strategies when animals are allowed a choice between both strategies. In the present study, we further explored the role of MRs in spatial and stimulus-response learning in two separate circular holeboard tasks using female mice with forebrain-specific MR deficiency and MR overexpression and their wildtype control littermates. In addition, we studied sex-specific effects using male and female MR-deficient mice. First, we found that MR-deficient compared to control littermates and MR-overexpressing mice display altered exploratory and searching behavior indicative of impaired acquisition of novel information. Second, female (but not male) MR-deficient mice were impaired in the spatial task, while MR-overexpressing female mice showed improved performance in the spatial task. Third, MR-deficient mice were also impaired in the stimulus-response task compared to controls and (in the case of females) MR-overexpressing mice. We conclude that MRs are important for coordinating the processing of information relevant for spatial as well as stimulus-response learning.

## Introduction

Corticosteroid hormones are secreted from the adrenals in an ultradian and circadian pattern, as well as in response to stressful experiences [1], [2]. Corticosteroid hormones regulate brain function via activation of high affinity mineralocorticoid receptors (MRs) and lower affinity glucocorticoid receptors (GRs) which are both expressed in brain areas which are crucial for learning and memory such as hippocampus, amygdala, prefrontal cortex and striatum [3]. Via activation of MRs and GRs, corticosteroid hormones promote behavioral adaptation to stressful experiences [1], [3]–[5]. MRs mediate initial behavioral responses to novel situations, and are required for adequate spatial and fear learning and memory processes [6]–[10]. Activation of GRs is crucial for consolidation of spatial and emotional information [6], [11]–[13].

Recently we demonstrated that corticosteroids are particularly important for the choice of strategies to solve navigational tasks. Different strategies such as spatial (hippocampus-dependent) or stimulus-response (striatum-dependent) strategies can be used to solve navigational tasks [14]–[16]. We designed a version of the circular hole board (CHB) task such that both spatial and stimulus-response strategies could be used to locate the exit hole (dual-solution task; [17]–[21]). When exposed to such a dual-solution task, the vast majority of male C57Bl/6j mice use a spatial strategy to navigate on a CHB [17]; to acquire a stimulus-response task, male mice have to overcome their natural tendency to use the spatial strategy [22]. Brief exposure to stress causes a shift from spatial towards stimulus-response strategies, an effect which is mediated via MRs [17].

In these studies, the CHB task was designed to allow animals to make a choice. However, it remains to be established to what extent activation of MRs determines stimulus-response and spatial strategies to solve navigational tasks per se, i.e. when animals do not have to make a choice.

In the present study we therefore studied the role of MRs in spatial (hippocampus-dependent) and stimulus-response (striatum-dependent) learning separately; i.e., we used, separately, the spatial and stimulus-response learning versions of the CHB task [22]. Moreover, to gain better insight into the role of brain MRs, we did not only examine the consequences of MR deficiency [7] but also investigated the other end of the spectrum, i.e. MR overexpression [8]. We hypothesized that MR overexpression will lead to improved performance in both tasks and might ease the shift from the spatial to the acquisition of the stimulus-response task, while the opposite is expected in the MR-deficient animals. We initially focused on *female* MR mutants, in view of the recently reported clear phenotype of female (but not male) MR-deficient mice in fear conditioning and the dual-solution CHB task [18], [19], [23]. Since we observed significant behavioral effects of MR deficiency in females, we next also examined the effects of MR deficiency in male mutants.

## Methods

### Animals

Male and female forebrain MR-deficient (MR^CaMKCre^; [7]) mice and their control littermates (MR^ flox/flox^)(n = 12 per group; males approximately 3 months and females approximately 4 months old) were bred in the animal facility of Leiden University. The MR^CaMKCre^ mice were obtained by breeding MR^flox/flox^ with MR^flox/wtCaMKCre^ mice from the German Cancer Research Center, Heidelberg, Germany. The conditional MR allele was generated in embryonic stem cells of 129Ola mice and CaMKCre transgene was injected in FVB/N mice [24]. The MR flox allele and the Cre transgene were backcrossed into C57Bl/6J mice. For a detailed description of the design and breeding of the MR^CaMKCre^ mice see [7]. In an additional experiment, female forebrain-specific MR-overexpressing transgenic (MR-Tg; [8]) mice and their control littermates were used (n = 19−20 per group; approximately 4 months old) that were bred in the animal facility of the University of Amsterdam. The MR-Tg mice were obtained from the Centre for Cardiovascular Science, Edinburgh, UK. A haemagglutinin (HA) epitope tag was inserted into the N-terminus of the full-length human MR cDNA. Transgenic mice were generated by injection of a CaMKIIa-HA-MR construct in C57Bl/6J CBA embryos (Babraham Institute, Cambridge, UK). For a detailed description of the design and breeding of the MR-Tg mice see [8]. After arrival, the mice were allowed to acclimatize to the animal facility for three weeks. Male and female MR-deficient mice were derived from 7 litters (2–6 mice per litter); 5 out of 7 litters contributed both MR^CaMKCre^ and controls (MR^flox/flox^). MR-overexpressing female mice were derived from 5 litters (3–4 mice per litter); we used MR-Tg mice and control littermates from all litters.

One week before the behavioral testing started, mice were moved to the experimental room (temperature: 20°C; humidity: 55% ±15), under a 12:12 hour light/dark cycle, lights on at 07:30 h) and single housed in Macrolon cages with sawdust bedding and with food and water *ad libitum*. Testing was carried out between 08:30 and 12:30 h. The experiments were approved by the committee on Animal Health and Care from Leiden University, The Netherlands, in accordance with the EC Council Directive of September 2010 (2010/63/EU).

### Apparatus

The circular hole board (CHB) is a revolvable grey round plate (Plexiglas; 110 cm in diameter; situated 1 m above the floor) with twelve holes at equal distances from each other, located 10 cm from the rim of the board. Holes are 5 cm in diameter and can be closed by a lid at a depth of 5 cm. Whether a hole is open or closed can only be detected if the mouse puts its head over the edge of the hole. An S-shaped tunnel (5 cm in diameter; 15 cm long) leads from the open exit hole to the home cage of the animal. Multiple distant cues in the room allow spatial orientation on the board [17], [18], [22].

### General Procedure

Each trial started by placing the mouse in a cylinder (Plexiglas; 25 cm high; 10 cm diameter) located at the center of the CHB. After 5 s the cylinder was lifted and the mouse could explore the board and exit through the open tunnel. If the mouse did not find the exit hole within 120 s, it was gently guided to the exit using a grid. The board was cleaned after each trial with 1% acetic acid solution to dissipate odor cues and rotated until another hole was at the position of the exit. The home cage was placed under the board at the position of the exit hole such that the mouse could not see the cage from the board.

### Timeline

**Figure 1:**
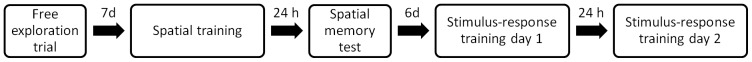
Experimental design of training and memory testing.

The experimental design of the experiment is schematically shown in Figure 1. We tested both male and female MR^CaMKCre^ mice, female MR-Tg mice and the control littermates of each group in two versions of the CHB task: a spatial task and a stimulus-response task. First, mice were given a free exploration trial (FET). One week later the spatial tasks started. Mice received six training trials (inter trial-interval 15 min), where only extra-maze spatial cues were available to locate the exit hole. One day after the spatial training, each mouse performed one spatial memory test trial with all holes closed. The stimulus-response task started one week after this spatial memory test and consisted of two subsequent days of each six training trials (again 15 min inter trial-interval), where an intra-maze stimulus (the bottle) marked the exit hole.

### Tunnel Training

One week before the behavioral experiments started the mice were weighed and trained to climb through a tunnel on every second day (three times in total). This familiarized mice with the task requirements.

### Free Exploration Trial

For the free exploration trial (FET) the mice were allowed to explore the CHB for 5 min. All holes were closed. At the end of the 5 min, the exit hole was opened and the animals were guided there by the experimenter. This exploration trial allowed to analyze exploratory behavior and general activity of the mice.

### The Spatial Task

One week after the FET, mice were given six successive training trials with a maximum of 120 s per trial. The location of the exit hole was always fixed relative to the distal extra-maze cues in the room. There were no proximal cues present, so the exit could only be found by using the extra-maze cues. This task was used to assess spatial learning. Twenty-four hours later, we tested long-term spatial memory.

### Spatial Memory Test

The mouse was placed on the CHB for two minutes with all holes closed. The behavior and movement pattern of the mouse allowed to analyze search strategy and spatial memory.

### Stimulus-response Task

In the stimulus-response (S-R) task, the position of the exit hole was marked by a bottle and varied from trial to trial in the same sequence for all mice. The position of the exit was never at the same location or a location adjacent to it within a six trial session. Furthermore, the exit hole location of the spatial task was not used as an exit hole position during the stimulus-response task. There were two subsequent days of six trials each. A trial lasted 120 s. The distal extra-maze cues were present, but only the proximal intra-maze cue (a transparent bottle filled with water; 0.5 L; 22 cm high; 5 cm diameter), located next to the exit hole, marked the exit. Therefore, the mice had to use a stimulus-response navigation strategy to locate the exit.

### Analysis of Behavior

Behavior was digitally recorded and analyzed with Ethovision XT 6.1 (Noldus Information Technology b.v., Wageningen, The Netherlands). This image analysis system sampled the position of the mouse 12.5 times per second. The CHB was virtually subdivided into subareas of special interest: center (start area), rim, zone of holes (area including all holes and the space between the holes, but excluding the center, middle and rim of the CHB) and four quadrants (covering three holes with the exit hole in the middle). We calculated preference values of the mice for the quadrant that contained the exit hole, and in the S-R task also for the quadrant that contained the exit hole of the previous trial. The following parameters were calculated by Ethovision: velocity (cm/s), distanced moved (cm), latency of first visit to exit hole (exit latency, s) (and to former exit hole for the S-R task), and latency to the quadrant of the exit hole (s) and duration in quadrant of the exit hole (s). The experimenter hand scored the number of holes visited (mouse puts at least its nose in the hole), rim dips (looking over the edge of the board), stretched attends, % perseveration (visiting the same hole twice in a row or with one other hole between the two holes) and % serial hole visits (visiting at least three adjacent holes in a row).

### Estrous Cycle

The stage of the estrous cycle was determined in female mice by vaginal cytology after each behavioral task. Using a plastic loop (inoculation loops 1 µl, Mediscan, Greiner Bio-one), a vaginal smear was obtained. The loop was dipped in water and then inserted into the vagina and gently rubbed against the vaginal wall. Cells were smeared on a glass slide in a drop of water. After air drying, the cells were stained with Giemsa (Sigma) for 10 minutes. The stage of the cycle was determined based on the presence or absence of nucleated epithelial, cornified epithelial and leukocyte cells. Proestrus: many cells with a nucleus and some epithelial cells; estrus: many epithelial cells and some cells with a nucleus; metestrus: some epithelial cells and many macrophages; diestrus: many macrophages and some cells with a nucleus. We did not encounter the metestrus stage.

### Statistical Analysis

Data are presented as mean ± SEM. Statistical analysis included one-way ANOVA, MANOVA, T-tests and General Linear Model repeated measures. Post hoc tests (Tukey) for multiple comparisons were used when appropriate. Reported p values are two-tailed and statistical significance was accepted for p<0.05. Statistical calculations were performed with IBM SPSS Statistics (version 20; SPSS Inc.; Chicago, IL). The numbers of female mice in the different stages of the estrous cycle were too low for test-statistics per stage. Therefore, we included the stage of the estrous cycle as a covariate in the one-way ANOVA and General Linear Model repeated measures analyses.

## Results

### Free Exploration Trial (FET)

#### MR^CaMKCre^ and MR-Tg female mice

MR^CaMKCre^ (deficient) female mice had a significantly longer latency to leave the center of the board when compared to their controls and also when compared to MR-Tg (overexpressing) female mice (8±1 vs 5±1; p = 0.039 and vs 3±1; p<0.0001, respectively). Furthermore, MR^CaMKCre^ female mice showed more perseveration than MR-Tg mice (20±3 vs. 11±2; p = 0.007). Exploratory behavior (% perseveration, % series, latency to leave the center, rim dips, rearing, stretched attends) and general activity (velocity, distance moved and number of holes visited) were comparable between MR-Tg mice and their control littermates (data not shown). Behavioral parameters were also comparable between control littermates of MR^CaMKCre^ and MR-Tg female mice.

#### MR^CaMKCre^ male

MR^CaMKCre^ males showed more perseveration and reared less than their controls (MR^CaMKCre^ vs controls, % perseveration: 27±5 vs 10±4; p = 0.018; rearing: 0.08±0.1 vs 1.25±0.5; p = 0.034).

### Spatial Learning

**Figure 2:**
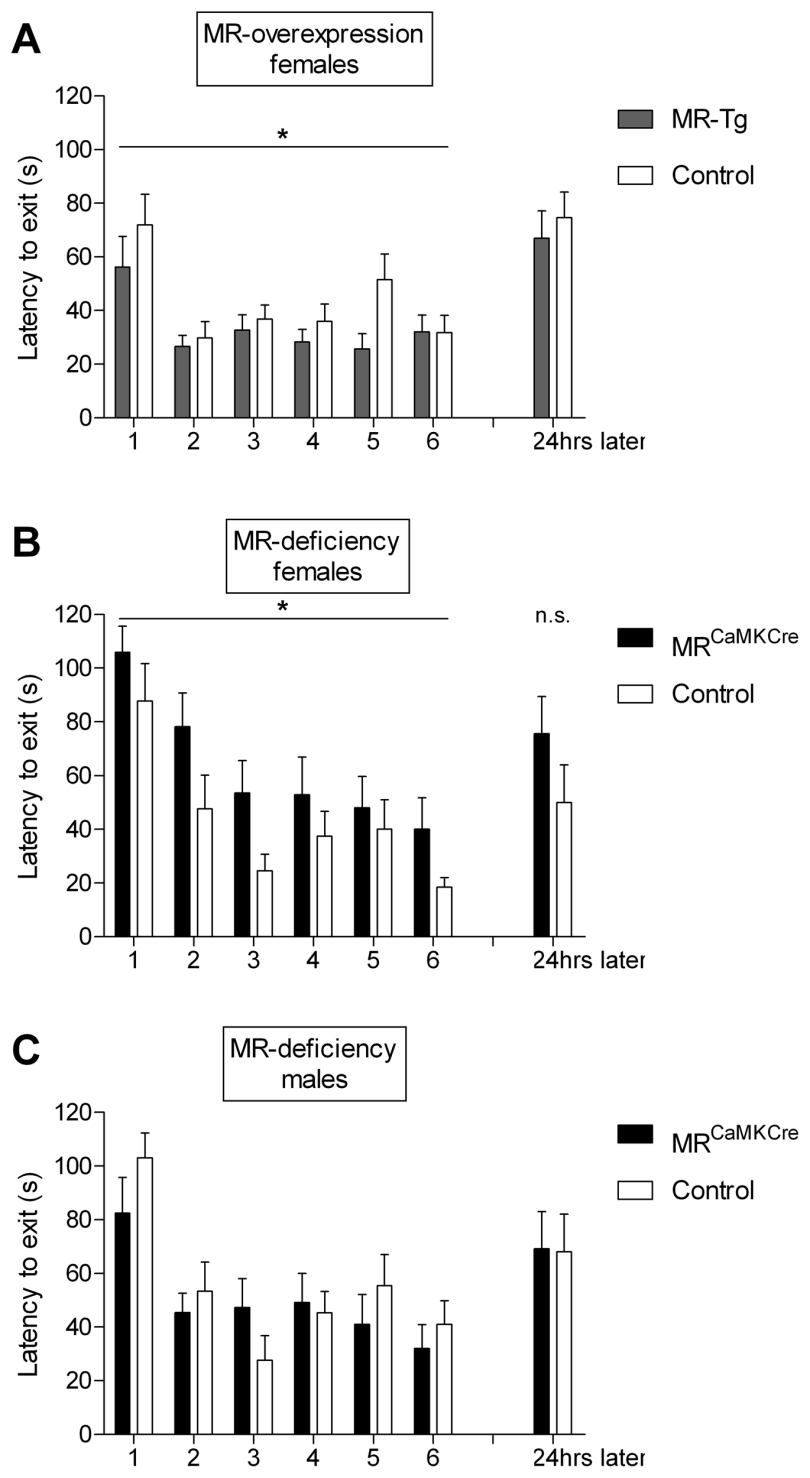
Spatial task: latency to the exit hole over six training trials and spatial memory test. A) Female MR-Tg mice (n = 19) take less time to find the exit hole than controls (n = 20). B) Female MR^CaMKCre^ mice take more time to find the exit hole than controls (n = 12 per group). C) Male MR^CaMKCre^ and control mice (n = 12 per group) have comparable latencies to locate the exit hole. Bars show mean ± SEM. *p<0.05 over trials, vs. control littermates. n.s. = not significant.

All groups acquired the task, as reflected by decreasing exit latencies over the trials (latency of first visit to the exit hole, MR-Tg females and their controls: F(5,185) = 6.338, p<0.0001; **Figure 2A**; MR^CaMKCre^ females and their controls: F(5,110) = 10.736, p<0.0001; **Figure 2B**; MR^CaMKCre^ males and their controls: F(5,110) = 10.011, p<0.0001; **Figure 2C**).

#### MR^CaMKCre^ and MR-Tg female mice

MR^CaMKCre^ females showed significantly impaired performance compared to their controls (exit latency: F(1,22) = 6.587, p = 0.019), while MR-Tg females out-performed the controls (F(1,37) = 4.893, p = 0.033). MR^CaMKCre^ females stayed longer in the center (F(1,22) = 4.986, p = 0.036) and had a lower velocity (F(1,22) = 7.335, p = 0.013) than their controls (**Table 1**) and than the MR-Tg mice (F(1,29) = 23.072, p<0.0001; **Table 1**). To figure out whether the impaired performance of the MR^CaMKCre^ compared to control females was caused by the longer time spent in the center, the exit latency was corrected for the latency to leave the center. When corrected, the MR^CaMKCre^ females still needed significantly more time to find the exit hole compared to their controls (F(1,22) = 4.941, p = 0.037; data not shown). Conversely, the MR-Tg females had a higher velocity during the trials than their controls (F(1,37) = 7.917, p = 0.008; **Table 1**). Other behavioral parameters were comparable between MR-Tg females and their controls. MR^CaMKCre^ female mice took longer to locate the exit than the MR-Tg females (F(1,29) = 16.090, p<0.0001), while controls of both groups had similar exit latencies (F(1,30) = 0.004, p = 0.949).

**Table 1:** Behavioral parameters (averaged over the day) recorded during the spatial training trials in MR-Tg and MR^CaMKCre^ mice and their control littermates.

	Females		Females		Males	
	MR-Tg	Control	MR^CaMKCre^	Control	MR^CaMKCre^	Control
Velocity (cm/s)	8±0*	7±1	5±0*	6±1	5±1	5±0
Latency to leave center (s)	3±0	3±0	10±3*^#^	5±1^$^	9±3	9±3

The spatial task consisted of six trials. Data represent mean ± SEM of all trials. For statistics a repeated measures ANOVA was used over the trials. Behavioral parameters that differ significantly; p<0.05: *vs same sex control littermates; ^#^female MR^CaMKCre^ vs MR-Tg; ^$^male vs female control littermates of MR^CaMKCre^ mice.

#### MR^CaMKCre^ males

The performance did not differ in any respect between MR^CaMKCre^ males and controls (**Figure 2C**; **Table 1**).

### Spatial Memory Test –24 Hours Later with Closed Exit

Latency to the exit hole was longer than in trial 6 the day before, but comparable between MR-Tg mice and their controls (**Figure 2A**) and between the MR^CaMKCre^ mice and their controls (**Figure 2B** (females) and **2C** (males)).

#### MR^CaMKCre^ and MR-Tg female mice

Both MR^CaMKCre^ female mice and control littermates spent more time in the exit (target) quadrant than in the other quadrants, but MR^CaMKCre^ females showed a trend for longer latency to the exit quadrant compared to their controls (56±15.4 vs 21±9.5; p = 0.070). MR^CaMKCre^ females spent less time in the holes zone and showed less perseveration than their controls (**Table 2**). Also the general activity parameters differed between MR^CaMKCre^ females and their controls. This is clear from the lower number of holes visited, shorter distance moved and a slower walking velocity in MR^CaMKCre^ females (**Table 2**). Parameters for exploration and general activity were comparable between MR-Tg mice and controls.

**Table 2:** Behavioral parameters recorded during the spatial memory test 24 hours after six spatial training trials in MR-Tg and MR^CaMKCre^ mice and their control littermates.

	Females		Females		Males	
Behavioral parameters	MR-Tg	Control	MR^CaMKCre^	Control	MR^CaMKCre^	Control
***General activity***						
Distance moved (cm)	741±49	741±65	264±29*^$#^	527±68	512±90	501±60
Velocity (cm/s)	6±1	6±1	2.5±0*^$#^	4.5±1	4±1	4±1
Total hole visits	9±1	9±1	3±1*^$#^	9±2	8±2	8±1
***Searching***						
Latency to exit quadrant (s)	21±7	35±9	56±15	21±10	64±15	30±11
Duration in exit quadrant (s)	50±6^∼^	40±7	59±1^∼$^	69±7^∼^	29±8	47±8^∼^
Average duration in other quadrants (s)	21±2	23±2	14±4^$^	13±3	26±3	21±3
Duration in holes zone (s)	41±5	35±3	23±8*^$#^	49±8	46±7	45±5
% Perseveration	16±3	13±3	5±3*^$#^	17±4	12±4	11±3

Data represent mean ± SEM. Behavioral parameters that differ significantly; p<0.05: *vs same sex control littermates; ^#^female MR^CaMKCre^ vs MR-Tg; ^$^male vs female MR^CaMKCre^; ^∼^duration in exit quadrant vs average duration in other quadrants.

#### MR^CaMKCre^ mice

The male MR^CaMKCre^ mice showed a trend for longer latency to the exit quadrant compared to their controls (males: 64±14.5 vs 30±10.9; p = 0.080). Furthermore, searching behavior also differed between MR^CaMKCre^ male mice and controls. MR^CaMKCre^ males showed a more evenly distributed searching pattern while their controls spent significantly more time in the exit quadrant than in the other quadrants (**Table 2**).

### Stimulus-response Learning

#### Performance

**Figure 3:**
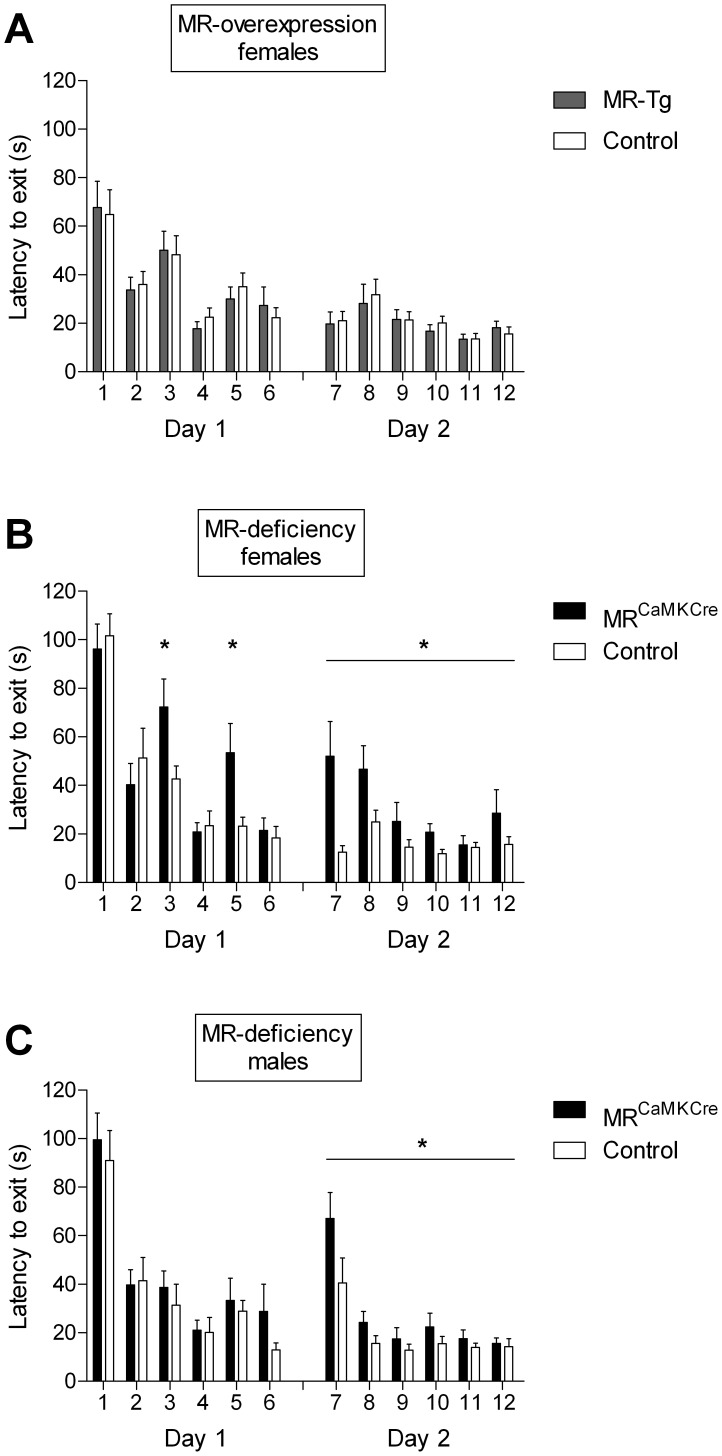
Stimulus-response (S-R) task: latency to the exit hole over six training trials on two days. A) Female MR-Tg mice and controls have short and similar latencies to the exit hole. B) Female MR^CaMKCre^ mice take more time to locate the exit than controls in trials 3 and 5 on day 1, and in all trials on day 2. C) Male MR^CaMKCre^ mice take more time to locate the exit than controls on day 2. Bars show mean ± SEM. *p<0.05 vs. control littermates.

One week after the spatial version of the CHB task, all animals were tested for stimulus-response (S-R) learning. All groups decreased their exit latencies over the trials (MR-Tg females and their controls: day 1: F(5,185) = 13.314, p<0.0001; day 2: F(5,110) = 3.725, p = 0.015; **Figure 3A**; MR^CaMKCre^ females and their controls: day 1: F(5,110) = 28.585, p<0.0001; day 2: F(5,110) = 3.406, p = 0.030; **Figure 3B**; MR^CaMKCre^ males and their controls: day 1: F(5,110) = 25.426, p<0.0001; day 2: F(5,110) = 16.049, p<0.0001; **Figure 3C**).

#### MR^CaMKCre^ and MR-Tg female mice

MR^CaMKCre^ mice showed impaired performance as evident from the longer latencies to the exit compared to their controls. On the first day of S-R training an interaction of genotype*trial was found (F(5,5) = 2.763, p = 0.040), with longer exit latencies in trials 3 and 5 in MR^CaMKCre^ females than controls (trial 3: p = 0.031; trial 5: p = 0.027). On the second day the MR^CaMKCre^ females had longer exit latencies than their controls over all trials (F(1,22) = 14.130, p = 0.001). In MR-Tg female mice exit latency was short and comparable to control littermates on both days. On the first day, exit latencies of MR-Tg and MR^CaMKCre^ as well as their controls were comparable. On the second day MR^CaMKCre^ females had longer latencies than MR-Tg females (F(1,29) = 8.441, p = 0.007), while their respective controls performed comparably.

#### MR^CaMKCre^ male mice

Also MR^CaMKCre^ male mice showed impaired performance on the second day of the S-R training compared to their controls (day 2: F(1,22) = 6.981, p = 0.015).

#### Short-term spatial memory in the S-R task

In every trial the exit hole was in a different position and always marked by the bottle. We interpret a visit to the position of the exit hole of the previous trial as an indication that a mouse applied a spatial strategy to locate the exit hole. Therefore, for every trial we measured the latency to the previous exit hole, the latency to the quadrant of the previous exit hole and the percentage of time spent in this quadrant (**Table 3**).

**Table 3:** Behavioral parameters (averaged over the day) recorded during the stimulus-response task in MR-Tg and MR^CaMKCre^ mice and their control littermates.

	Females		Females		Males	
	MR-Tg	Control	MR^CaMKCre^	Control	MR^CaMKCre^	Control
***Day 1***						
Velocity (cm/s)	8±0	8±0	5±1*^$^	8±1	7±1	8±1
Distance moved (cm)	367±64	380±59	296±47	287±48	313±64	253±46
Total hole visits	6±1	6±1	4±1*	5±1	5±1	5±1
%Perseveration	8±3*	4±2	7±4	6±2	3±2	3±3
Latency to previous exit quadrant (s)	48±12	46±12	72±14*	47±14	58±15	51±15
Duration in previous exit quadrant (s)	18±4	18±4	13±5*	23±5	14±2*	21±3
***Day 2***						
Velocity (cm/s)	9±1	9±1	7±1*	9±1	7±1	8±1
Distance moved (cm)	213±31*	253±38	249±51	213±49	245±46	207±38
Total hole visits	3±1	4±1	3±1	3±1	3±1	3±1
%Perseveration	5±2*	5±2	3±2	2±2	3±2	3±2
Latency to previous exit quadrant (s)	51±12	45±11	48±13	48±14	55±16	61±16
Duration in previous exit quadrant (s)	14±4	12±3	17±5	22±4^$^	14±2	10±1

Both days of the stimulus-response task consisted of six trials. Data represent mean ± SEM of all trials of one day. For statistics a repeated measures ANOVA was used over the trials of one day. Behavioral parameters that differ significantly; p<0.05: *vs same sex control littermates; ^#^female MR^CaMKCre^ vs MR-Tg; ^$^male vs female MR^CaMKCre^.

On the first day of the S-R training, the time spent in the quadrant of the previous exit was significantly shorter for MR^CaMKCre^ males and females than for the control littermates (males: F(1,22) = 7.615, p = 0.011; females: F(1,22) = 9.356, p = 0.006; **Table 3**). Latency to the quadrant of the previous exit was longer for MR^CaMKCre^ female mice than for their control littermates (F(1,22) = 7.953, p = 0.010; **Table 3**) with no difference between MR^CaMKCre^ male mice and controls. On the second day, latency to the previous exit hole was comparable between the MR^CaMKCre^ mice and controls in both males and females. Latency to the exit hole and exit quadrant of the previous trial were also comparable between MR-Tg females and controls.

#### Long-term spatial memory in the S-R task

Before training in the S-R task, mice had received spatial training, with the exit in a fixed location in relation to the extra maze cues. These cues were still available in the S-R task, however now the exit was variable marked by a bottle, irrespective of spatial cues. We expected that spatial memory might influence the acquisition of the S-R task. Therefore, we measured the latency to the exit hole of the spatial task during the first trial of the first S-R training day.

Latency to the spatial exit was comparable between MR-Tg females and their control littermates. MR^CaMKCre^ females took longer to reach the quadrant of the spatial exit (p = 0.015) and spent less time in this quadrant (p = 0.018) while the latency to the spatial exit hole was similar to their controls (**Table 4**). MR^CaMKCre^ males took significantly longer to reach the quadrant of the spatial exit hole (p = 0.042), spent less time in this quadrant (p = 0.043) and showed a trend of longer latency to the spatial exit hole than their controls (p = 0.095) (**Table 4**).

**Table 4:** Spatial exit memory during the first trial of the stimulus-response task on day 1 (one week after the spatial memory test) in MR-Tg and MR^CaMKCre^ mice and their control littermates.

	Females		Females		Males	
Behavioral parameters	MR-Tg	Control	MR^CaMKCre^	Control	MR^CaMKCre^	Control
Latency to spatial exit hole (s)	68±12	82±11	88±14	71±13	96±11	64±15
Latency to spatial exit quadrant (s)	38±12^#^	38±11	77±16*	28±10	73±13*	36±11
Duration in spatial exit quadrant (% of time on CHB)	34±6	36±6	20±9*	51±8	23±6*	45±8

Data represent mean ± SEM. Behavioral parameters that differ significantly; p<0.05: *vs same sex control littermates; ^#^female MR^CaMKCre^ vs MR-Tg.

#### Searching strategies and general activity in S-R trials

Searching strategies were defined as a way to find the exit hole, expressed by the order of holes visited. A serial hole visit was defined as visiting at least three adjacent holes in a row. Perseveration was defined as visiting the same hole twice in a row or with one other hole between the two holes.

#### MR^CaMKCre^ and MR-Tg female mice

On the first day, MR^CaMKCre^ female mice visited less holes in a serial manner than their control littermates (% series: F(1,22) = 11.509, p = 0.003), while on the second day the genotypes had similar percentages of serial hole visits (**Table 5**). Both MR-Tg genotypes had a similar percentage of serial hole visits (**Table 5**). MR^CaMKCre^ female mice moved slower than their controls on both days (velocity; day 1: F(1,22) = 14.592, p = 0.001; day 2: F(1,22) = 6.083, p = 0.022) and visited less holes on the first day (F(1,22) = 8.691, p = 0.007; **Table 3**). Velocity on both days and distance walked on the first day were similar between MR-Tg female mice and controls. On the second day, the MR-Tg female mice walked less than the controls (distance; F(1,37) = 5.000, p = 0.031; **Table 3**). During both days of the stimulus-response task, MR-Tg female mice showed more perseveration than the controls (day 1: F(1,37) = 4.594, p = 0.039; day 2: F(1,37) = 8.390, p = 0.006; **Table 3**).

**Table 5:** Percentage of serial hole visits averaged over the trials of the two days of the stimulus-response task.

	Females		Females		Males	
Stimulus-response task	MR-Tg	Control	MR^CaMKCre^	Control	MR^CaMKCre^	Control
Day 1	19±6	18±5	14±7	27±8*	18±7	38±9*
Day 2	11±5	14±5	13±6	16±6	11±6	11±6

Both days of the stimulus-response task consisted of six trials. Data represent mean ± SEM of all trials of one day. For statistics a repeated measures ANOVA was used over the trials of one day.

* Significantly different between genotypes of the same group over the day; p<0.05.

#### MR^CaMKCre^ male mice

Searching strategies were different between the genotypes in MR^CaMKCre^ male mice. On the first day, MR^CaMKCre^ males visited less holes in a serial manner than their control littermates (% series: F(1,22) = 19.489, p<0.0001), while on the second day the genotypes had similar percentages of serial hole visits (**Table 5**). In contrast to the females, general activity was comparable between genotypes for MR^CaMKCre^ male mice.

### Behavior and the Estrous Cycle

We determined the stage of the estrous cycle in female mice by vaginal cytology after each behavioral task. We did not encounter the metestrus stage. The numbers of MR^CaMKCre^ female mice and controls in the proestrus stage in all behavioral tasks were too low for test-statistics. The numbers of MR-Tg females and controls in the proestrus stage in the FET were too low for test-statistics. The estrus stage was well represented in all behavioral tasks in all groups. Given this uneven distribution, we opted for including the stage of the estrous cycle as a covariate in the one-way ANOVA and General Linear Model repeated measures analyses. These analyses did not support a significant contribution of estrous cycle on performance.

## Discussion

To further delineate the role of MRs in spatial and stimulus-response learning we subjected mice either lacking or overexpressing MRs in the forebrain to a spatial and stimulus-response learning task. Solving these tasks requires hippocampal and dorsal striatum memory systems, respectively. Spatial learning is the predominant form of learning in male rodents; acquisition of stimulus-response learning requires more training trials [22], [25]. In previous studies we had tested performance and the use of memory systems of mice with manipulated MR expression in a dual-solution CHB task. Pharmacological blockade of MR as well as MR deficiency led to deterioration of performance in naïve non-stressed male mice [17]–[19]. However, in this design the selected strategies were mutually exclusive (animals had to choose between the two strategies), so that we did not know to what extent MR expression specifically influences the two systems. Thus, the impairment in performance could have been the result of a dysfunction of the hippocampus, a dysfunction of the dorsal striatum or both, or the coordination in the behavior-controlling network.

We report here that the expression of MRs is critical for both memory systems. Thus, forebrain MR-deficient female mice showed both an impaired spatial and stimulus-response learning, while MR overexpression resulted in opposite effects, i.e. improved spatial performance while stimulus-response learning was not improved, probably due to a ceiling effect. Performance deficits in both tasks were more strongly expressed in MR-deficient female than male mice, revealing and supporting a sex-dependent effect of MR deficiency. The opposite results of MR deficiency and MR overexpression confirm and substantiate the impact of MR on acquisition of novel information, not only for spatial but also for stimulus-response learning.

### Behaviour in a Novel Environment and Learning

Being exposed to the CHB after a life in a small cage with conspecifics is a challenge for the mouse. Exploration is the natural response. We and others [6], [18], [26], [27], have shown that activation or blockade of MRs in rats and mice alters the exploration pattern and behavioral flexibility but not general activity measures. We here show that MR-overexpressing female mice showed an exploration pattern comparable to their control littermates, while MR-deficient male and female mice showed different exploratory behavior compared to controls without alterations in general activity: persistent revisits of holes in the male mice and longer time spent in the center before exploring the CHB in the female mice. These behaviours appear to be characteristic for MR^CaMKCre^ mice, as they are in line with behavioral responses previously seen when MR-deficient mice were exposed to conditions of novelty [7], [10], [18], [19]. After acute pharmacological inhibition of MR in rats goal-directed search strategies in a water maze task were absent [6]. In contrast, selectively activating MRs in adrenalectomized rats normalized the exploration pattern [26]. In line with MR effects in rodents, the few studies that were done in humans with pharmacological blockade of MR reported a deficit of selective attention and impairment of working memory [28], [29] indicating difficulties in the acquisition of novel information.

Overall, behavior in the absence of functional MRs might represent a restriction of behavioral flexibility [18], while selective activation of MR allows adequate explorative behavior and adaptation.

We reproduced an earlier finding [19] that MR^CaMKCre^ female mice remain longer in the center during spatial training. Conversely, MR-overexpressing mice displayed more entries into the central area of an open field and had shorter latencies entering the light compartment of a light/dark task, which was labeled by others as reduced anxiety-related behavior [8]. This suggests that staying longer in the center as reported for the MR^CaMKCre^ mice is due to enhanced anxiety. Yet, unconditioned anxiety was not observed in MR^CaMKCre^ mice [7], [10], [18], [23]. The behavior of MR^CaMKCre^ mice may therefore also point to a different coping style.

Even when subtracting the time in center from the overall latency to the exit hole, the latencies of MR^CaMKCre^ mice were longer than in control mice. It is likely that the different exploration pattern of MR-deficient mice seen during the initial exposure to the CHB underlies or at least contributes to their impaired performance in the spatial and stimulus-response tasks. Processing of information from the environment is depending on the MR function, as shown by behavior 24 hrs after spatial learning and during the stimulus- response task.

### Novel Situation of Closed Exit Hole 24 Hrs after Spatial Learning

Exposing the mice 24 hrs after six spatial trials to the CHB with the exit hole closed can be compared to a probe (free exploration trial) in the water maze. Latency to the exit hole reflects retention of spatial memory, but their behavior is also the response to this novel situation with no exit available. Similar latency to exit indicates that retention is comparable between the groups. Time to leave the center was comparable between the groups.

Other measures provide a wealth of information. In the water maze task, the time spent in quadrant is mainly used to demonstrate the strength of spatial memory. Earlier studies in the water maze showed that MR-overexpressing male mice spent significantly more time during the probe trial in the target quadrant than control mice [8], [27]. Is this strong memory or perseveration which could be another kind of coping with novelty? In the present study, control mice of all groups, spent most time in the target quadrant as do MR^CaMKCre^ female and MR-Tg female mice. MR^CaMKCre^ male mice spent a comparable amount of time in all quadrants. Latency to the target quadrant was shorter in all control groups and MR-Tg mice than in MR^CaMKCre^ mice. Moreover, MR^CaMKCre^ female mice had the lowest number of hole visits, spent the least time in the zone with holes and moved the shortest distance compared to all groups. This may reflect a similar difference in coping style as observed on the first day.

We tentatively conclude that spatial memory - when expressed by latencies - is not affected by MR. However, coping with a novel situation seems to depend on MR.

### MRs Involved in Stimulus-response Learning

In rodents, stimulus-response learning takes longer and requires more training than spatial learning: they have to overcome their natural tendency to use a spatial strategy [22], [25]. We expected that training the mice in the spatial version of the CHB task preceding training in the stimulus-response task might even amplify the difficulty in acquiring stimulus-response learning. On the other hand, due to prior training, the environment is familiar and mice have learned that there is an exit hole. With this prior experience they now have to learn that the exit hole is marked by an intramaze cue, the bottle, positioned at a different location every trial. Thus, again, mice had to be flexible and adapt to a novel situation. Will mice express a spatial bias to the fixed location of the exit hole learned during spatial training?

As expected, the control littermates of the MR^CaMKCre^ male and female mice and the MR-Tg mice and their control littermates showed a spatial tendency (more time in the spatial exit quadrant) in the first trial of the stimulus-response task. On the first day of training, controls of the MR^CaMKCre^ mice used more serial searches and switched to a preference of the stimulus-response strategy on the second day. MR-Tg female mice showed more perseverations but a comparable low percentage of serial searches as their control littermates.

The spatial tendency was absent in MR^CaMKCre^ male and female mice, which could e.g. be explained by their poorer earlier performance in the spatial task or by a higher propensity to switch to the stimulus-response strategy. However, we can rule out the latter possibility because we could not detect a stimulus-response strategy on either day. The MR^CaMKCre^ mice simply took more time to solve the task. In contrast, MR-Tg mice were as fast as their control littermates. Since mice of all control groups perform well and the latency to the exit is short, we suggest a ceiling effect in performance, which may explain why we could not detect further improvement of MR-overexpressing mice.

Performance of MR-deficient mice was impaired compared to MR-overexpressing mice, but comparable to their controls during that first stimulus-response trial. As revealed by analysis over the trials of the first day of stimulus-response training, the performance of control mice appears to be guided by spatial tendency, which was absent in the MR-deficient mice. Therefore, we propose that MR deficiency exerts a more general effect on behavior by inhibiting or delaying the adaptation to novel requirements. The current behavioral set-up did not allow to distinguish between a strong spatial tendency and reduced behavioral flexibility, as both will result in long latencies in the first stimulus-response trial after spatial training.

Overall, the MR^CaMKCre^ mice seem to lack the high degree of behavioral flexibility that is required for optimal performance in the stimulus-response task.

### Influence of Task-dependent Characteristics on Behaviour

Previous studies using the dual-solution CHB and water maze tasks reported impaired performance, predominantly delayed learning, in MR^CaMKCre^ male mice [7], [18]. In the present study, MR^CaMKCre^ male mice performed comparable to control mice. Task-specific characteristics might have influenced the performance in several ways. First, for mice the water maze is a more stressful task than a dry land maze task such as the CHB [30], [31]. Acute stress impaired the performance of control mice in the dual-solution and spatial CHB task [17], [22]. Interestingly, acute stress did not further impair performance of MR^CaMKCre^ male mice in the dual-solution CHB task [18]. Second, the dual-solution version of the CHB task provides both intramaze and spatial cues. Since more cues are available, this requires a fine tuned coordination of hippocampal and dorsal striatal memory systems. The coordination of memory systems might be affected by MR deficiency, resulting in delayed learning.

In contrast to the MR-deficient mice, MR-overexpressing male mice showed no difference in spatial learning in water maze and Y-maze tasks [8], [27]. Moreover, a rather selective method, namely viral-mediated overexpression of MR in the hippocampus in male rats caused no differences in spatial performance in a water maze task [32]. The findings in MR-overexpressing mice suggest that the behavioral parameters of the tasks might not be sensitive enough to measure MR effects. Alternatively, we may conclude that MR manipulations do not directly affect the memory process.

### Are Female Mice More Sensitive than Males to Changing Environments?

Male C57Bl/6j mice and control littermates of MR^CaMKCre^ male mice solve the dual-solution CHB task by using a spatial strategy, while female C57Bl/6j and control littermates of MR^CaMKCre^ female mice use both, spatial and stimulus-response strategies [18]–[21]. MR deficiency led to impaired spatial performance in MR^CaMKCre^ female mice, while the effect was less apparent in MR^CaMKCre^ male mice. Similar results were found in other learning tasks. For example, MR^CaMKCre^ female mice were unable to extinguish the contextual fear memory and could not discriminate between cue and context episodes of the task, while no effects of MR ablation were found in MR^CaMKCre^ male mice [23]. In a radial arm maze task, MR^CaMKCre^ female mice made more errors than males [7]. Sex-dependent differences in performance were aggravated by acute stress: male mice switched from a spatial to a stimulus-response strategy, while female mice switched to a spatial strategy [17], [33]. These observations are in line with our present findings and underline that using subjects of both sexes increases the likelihood to detect effects of the experimental manipulation. In general, variations in MR appear to affect females more explicitly than males.

In addition to the overall sex-dependent differences, the phase of the estrous cycle may influence the behavior of female mice [19], [20], [34]. Recently we reported a specific interaction between MR and female sex hormones [19]. MR^CaMKCre^ females showed impaired performance in the dual-solution CHB task specifically in the proestrus and estrus phase of the cycle. The phase of the estrous cycle had no effect on performance of C57Bl/6j and control littermates of MR^CaMKCre^ female mice [19]. Due to the relatively low numbers per cycle stage, we could not incorporate the data on the estrus phases in the current study, which is a limitation. However, introducing the cycle stage as a co-variate in our analysis did not affect the outcome.

### Two Receptors for Adaptive Behavior: MR and GR

Adaptive behavior depends on balanced MR and GR activation [1], [35]. As documented previously [6], [7], [10], [18] and extended by our current observations, MRs modulate the behavioral response pattern in novel situations. We cannot estimate the number of MRs that is necessary to induce the changes observed in MR-overexpressing mice. Lai et al (2007) report a brain-site-dependent increase of MR mRNA of 4 to 10 times in MR-Tg mice.

MR deficiency in the forebrain affects the regulation of the hypothalamic-pituitary-adrenal (HPA) axis. Basal corticosterone levels of MR-deficient mice are either comparable to controls [7], [10], but also elevated basal corticosterone levels have been reported [18]. A fast increase of corticosterone in response to stress indicates the lack of MR-dependent inhibitory control of the HPA axis and apparently more efficient negative feedback can be deduced from lower corticosterone measured directly after training in a circular holeboard task [18]. Elevated basal corticosterone in MR-deficient mice might reflect the sensitivity of the HPA axis to subtle environmental changes, which are under inhibitory control of MR in control mice. MR-overexpressing mice and their control littermates had comparable basal corticosterone levels and showed a comparable corticosterone response to restraint stress [8]. Like previously reported, we expect that the mice in the present study also show this initial MR-dependent disinhibition the HPA axis resulting in elevated concentrations of corticosterone.

If and how these different-to-control corticosterone concentrations in MR-mutant mice affect and act via GRs: we don’t know yet. The characteristics and action of GR (low affinity, fast feedback) indicate an involvement of GR in MR-deficient mice in a time domain different from control mice. GRs are involved in HPA axis regulation and stress effects on cognition, specifically supporting memory consolidation [6], [11]–[13]. GR mRNA is increased in the hippocampus of MR^CaMKCre^ mice; however their memory is not improved [18]. GR mRNA and basal corticosterone levels in MR-Tg mice are comparable to controls, as is their memory [8]. Therefore, we cannot exclude that deficits in spatial and stimulus-response performance in the MR-deficient mice are linked to increased circulating basal corticosterone levels that may act via GR.

### Memory Systems, Glucocorticoids and MR: Human Studies

Previous research showed that glucocorticoids are involved in the stress-induced shift from hippocampus-dependent to dorsal striatum-dependent learning. In human studies, this role of glucocorticoids was extended from navigational to other forms of learning [21], [36], [37]. Recently, Schwabe et al. suggested that the relationship between glucocorticoid concentrations and the use of different memory systems may not be linear but more likely in the shape of an inverted u-shaped curve [37], [38]. This reasoning is based on findings that humans exhibiting high stress-induced cortisol responses used more stimulus-response learning [39], while spatial learners had higher basal cortisol concentrations [40] and pharmacological elevations of cortisol led to use of more spatial learning [41]. Schwabe et al. discussed that functioning of the hippocampus and dorsal striatum may be affected differently by low, moderate and high levels of glucocorticoids, and thus, allowing different memory systems to be in control of behavior [37], [38]. Also here we might deal with a differential contribution of MR and GR to the behavioral effects which have not been entangled yet.

Recently, blockade of MR was reported to prevent the stress-induced shift from hippocampal towards dorsal striatum-dependent learning in a classification task in humans, underlining the importance of MR for the use of multiple memory systems [42]. Furthermore, stress-induced facilitation of inhibitory control in a stop-signal task in humans was reported to depend on MR functioning, indicating that MRs are important for the balance between inhibition and excitation that underlies adaptive behavior [43]. Although the use of MR (and GR antagonists) contributes to the understanding of the function of either receptor, we are still confronted by the fact that the blockade of MR (or deficiency of MR) increases corticosterone concentrations that should allow the activation of GR.

In summary: our current study demonstrates that MRs are relevant for spatial as well as stimulus-response learning. Deficits in both tasks were more strongly expressed in MR-deficient female than male mice, revealing and supporting a sex-dependent effect. We suggest that the common nominator of MR effects can be represented as behavioral flexibility which requires a critical balance between inhibitory and excitatory systems. The consequences of corticosteroid actions via MR can be observed at different levels, e.g., the switch between memory systems, strategies, selective attention, performance. In this manner MRs influence and coordinate the processing of information particularly under novel conditions, which is fundamental for behavioral adaptation.
